# Transduction of Human T Cells with a Novel T-Cell Receptor Confers Anti-HCV Reactivity

**DOI:** 10.1371/journal.ppat.1001018

**Published:** 2010-07-29

**Authors:** Yi Zhang, Yeuying Liu, Kelly M. Moxley, Lucy Golden-Mason, Michael G. Hughes, Tongxin Liu, Mirjam H. M. Heemskerk, Hugo R. Rosen, Michael I. Nishimura

**Affiliations:** 1 Division of Transplantation, Department of Surgery, Medical University of South Carolina, Hollings Cancer Center, Charleston, South Carolina, United States of America; 2 Division of Gastroenterology & Hepatology, Hepatitis C Center & Department of Medicine, University of Colorado Health Sciences Center, Denver, Colorado, United States of America; 3 Laboratory of Experimental Hematology, Department of Hematology, Leiden University Medical Center Leiden, the Netherlands; 4 Division of General Surgery, Department of Surgery, Medical University of South Carolina, Hollings Cancer Center, Charleston, South Carolina, United States of America; University of Washington, United States of America

## Abstract

Hepatitis C Virus (HCV) is a major public health concern, with no effective vaccines currently available and 3% of the world's population being infected. Despite the existence of both B- and T-cell immunity in HCV-infected patients, chronic viral infection and HCV-related malignancies progress. Here we report the identification of a novel HCV TCR from an HLA-A2-restricted, HCV NS3:1073–1081-reactive CTL clone isolated from a patient with chronic HCV infection. We characterized this HCV TCR by expressing it in human T cells and analyzed the function of the resulting HCV TCR-transduced cells. Our results indicate that both the HCV TCR-transduced CD4^+^ and CD8^+^ T cells recognized the HCV NS3:1073–1081 peptide-loaded targets and HCV^+^ hepatocellular carcinoma cells (HCC) in a polyfunctional manner with cytokine (IFN-γ, IL-2, and TNF-α) production as well as cytotoxicity. Tumor cell recognition by HCV TCR transduced CD8^−^ Jurkat cells and CD4^+^ PBL-derived T cells indicated this TCR was CD8-independent, a property consistent with other high affinity TCRs. HCV TCR-transduced T cells may be promising for the treatment of patients with chronic HCV infections.

## Author Summary

Hepatitis C Virus (HCV) is a major public health concern with a large number of individuals infected (3% world wide). Currently, there is no effective vaccine available to prevent HCV infection and the treatment is effective in less than half of all patients. Therefore, many patients have long term infections that lead to severe liver damage or liver cancer. It has been shown that some HCV infected patients can eliminate the virus and the host immune system is involved. The problem is most people do not have the capacity to fight their HCV infection. We have developed a gene therapy based approach where a patient's own immune cells can be made to recognize cells expressing HCV genes. This can be accomplished regardless of his or her natural capacity to fight their HCV infection. This manuscript describes how normal immune cells can be genetically altered to recognize cells expressing HCV proteins and characterizes their reactivity and sensitivity to antigen stimulation.

## Introduction

Hepatitis C Virus (HCV) infection is a major public health concern with approximately 3% of the world's population being infected [1]. Unfortunately, 70–80% of infected individuals are unable to clear the virus, resulting in a chronic infection with the potential for developing severe liver diseases such as cirrhosis and hepatocellular carcinoma (HCC) [2], [3]. These liver diseases are the major indication for liver transplantation in the US and Europe [4], [5]. The combination therapy of interferon-α and ribavirin is used to treat HCV infections with limited success [6]. The development of preventative and therapeutic vaccines has been hindered by a lack of relevant animal models to study HCV viral replication and disease progression *in vivo*.

Both cellular and humoral immunity exists against HCV proteins in HCV-infected individuals [7]. However, not all HCV-infected patients can mount an effective anti-HCV immune response leading to the reduction of the viral load [8], [9]. There is evidence that demonstrates that the HCV genome mutates rapidly suggesting that mutations in T-cell and B-cell epitopes lead to immune escape variants which may be the main reason for HCV persistence in chronically infected patients [10]–[12]. Therefore, until better immune-based strategies are developed, immune therapy will have limited benefit for HCV-infected patients.

An approach has been described in which retroviral vectors encoding T-cell receptor (TCR) genes are used to redirect the specificity of normal peripheral blood lymphocyte (PBL)-derived T cells to recognize the melanoma associated antigen, MART-1 [13], [14]. Subsequently, this approach has been extended to other tumor antigens and viruses [15]–[28]. In fact, three phase I/II clinical trials using this approach to treat melanoma have been reported [29]–[31]. In two of these studies, no serious adverse events were observed and a few objective clinical responses were reported [29], [30]. However, the third study reported an increase in the frequency of clinical responses but a few patients experienced adverse events [31]. With TCR gene transfer becoming a reality for cancer patients, it opens the possibility for engineering a patient's own T cells to recognize their HCV virus-infected cells, regardless of their immune status.

It has also been well known that the HCV genome contains several regions and it is genetically unstable and mutates readily. The high variation of HCV is used to produce escape mutants that can sneak past the immune response of the host. The variants also play a significant role in the progression of virus infection due to resistance to immunotherapy. We have previously identified HCV NS3:1406–1415 reactive T cells that express high-affinity TCRs [24], [32]. In the current study, we cloned novel HCV TCR genes from an HLA-A2-restricted, HCV NS3:1073–1081-reactive, T-cell clone isolated from a patient with chronic HCV infection. This is an important epitope since it is frequently the immunodominant epitope targeted by anti-HCV CTL in HCV infected patients [33]. It also shares sequence homology with a peptide from the influenza A virus and CTL have been shown to crossreact with both peptides [34]. And finally, it is often mutated in HCV immune escape variants making it likely to be an important target in the anti-HCV immune response [35]. For the first time, we demonstrate that a recombinant retroviral vector encoding the HCV NS3:1073–1081 TCR could efficiently transduced both CD4^+^ and CD8^+^ T cells. Functional analysis demonstrates that the HCV TCR-transduced both CD4^+^ and CD8^+^ T cells produce interferon-γ, TNF-α, and to a lesser extent, IL-2 when stimulated with the peptide-loaded targets or HCV^+^ hepatoma cells. Our results indicate that an HCV TCR can engineer antigen reactive CD4^+^ and CD8^+^ T cells, raising the possibility that we can provide any HCV patient with a source of autologous HCV-reactive T effector and helper cells which have been implicated in eradicating HCV infections.

## Results

### Identification of the TCR Genes Used by an HCV Reactive T Cell Clone

**Figure 1:**
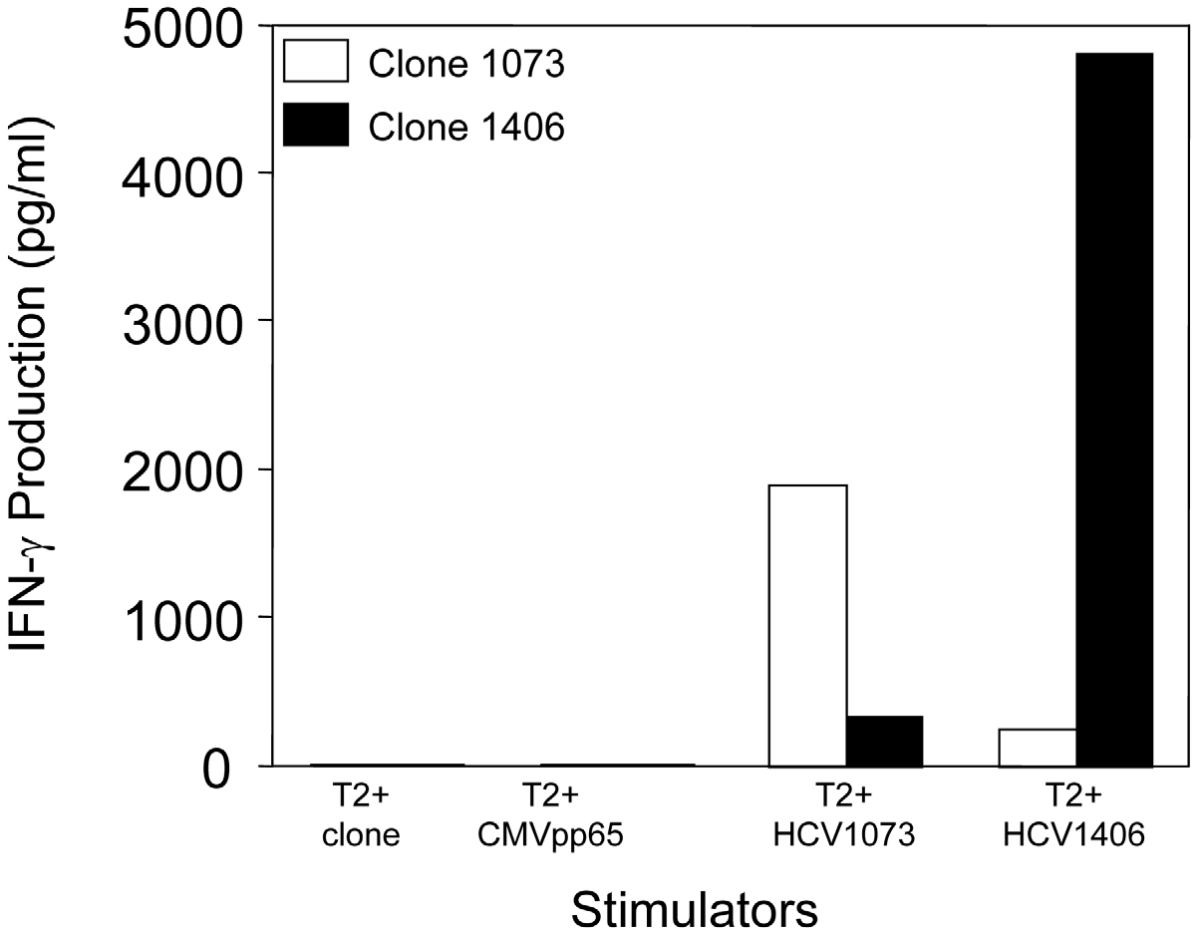
Antigen recognition by HCV T-cell clone 1073. The specificity of HCV-reactive T cell clones was assessed using interferon-γ release assays. T2 cells were incubated for 2 hr with 5 µg/ml of HCV NS3 1073–1081, HCV NS3:1406–1415, or CMVpp65 peptide. Peptide-loaded T2 cells were incubated for 20 hr in microwells with the HCV T cell clone 1073 (white bars). As a specificity control, an HCV NS3:1406–1415 T cell clone (black bars) was included in the assay. The amount of interferon-γ produced was measured by ELISA.

An HCV NS3:1073–1081-reactive CTL clone was isolated from the blood of a patient with a chronic HCV infection by limiting dilution cloning. The T cell clone was analyzed for antigen recognition in cytokine release assays. As shown in Figure 1, the T cell clone secreted significant amounts of interferon-γ when stimulated with T2 cells loaded with the HCV NS3:1073–1081 peptide but not the control CMVpp65 or HCV NS3:1406–1415 peptides. These results indicate that the T cells isolated from the patient with a chronic HCV infection were reactive with the HCV NS3:1073–1081 antigen.

**Figure 2:**
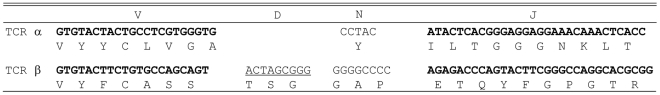
Junctional sequences of the TCR α chain and the TCR β chain identified from HCV NS3:1073–1081 clone. TCR analysis revealed that the HCV NS3:1073–1081 clone expressed a single TCR α chain (AV20s1) and a single TCR β chain (BV13s6). The germline V genes and J regions (and D region for the β chain) are shown for each TCR chain. The unique N regions in the CDR3 region of each chain are listed.

The HCV NS3:1073–1081 TCR α and β chains from the HCV NS3:1073–1081-reactive, T cell clone were identified as described [36]. The germline V genes, J regions, and the unique CDR3 region sequences for each TCR chain are shown in Figure 2. DNA sequence analysis of random 5′ RACE TCR α chain cDNA clones revealed the HCV NS3:1073–1081 T cell clone expressed a single TCR α chain consisting of AV20s1/AJ10/AC. This TCR α chain was in-frame and contained all of the landmarks consistent with a functional TCR α chain. The TCR β chain was identified by RT-PCR using a panel of TCR BV subfamily specific primers as described [37]. The only primer that amplified a fragment of the predicted size was the BV13 primer suggesting that the TCR expressed by the HCV NS3:1073–1081 reactive T cell clone used member of the BV13 subfamily (data not shown). DNA sequence analysis of that PCR fragment revealed that the TCR β chain consisted of BV13s6/BJ2s1/BJ2s7/BC2. Like the TCR α chain, the DNA sequence of the TCR β chain indicated it was in-frame and contained all of the features consistent with a functional TCR β chain. The identification of the TCR β chain being BV13 was confirmed by immunofluorescence staining with an anti-Vβ13s6 mAb (data not shown). Thus, the HCV NS3:1073–1081 T cell clone expresses an AV20s1/BV13s6 TCR.

### Construction of an HLA-A2^+^ HCC Cell Line Expressing the HCV NS3:1073–1081 Epitope

**Figure 3:**
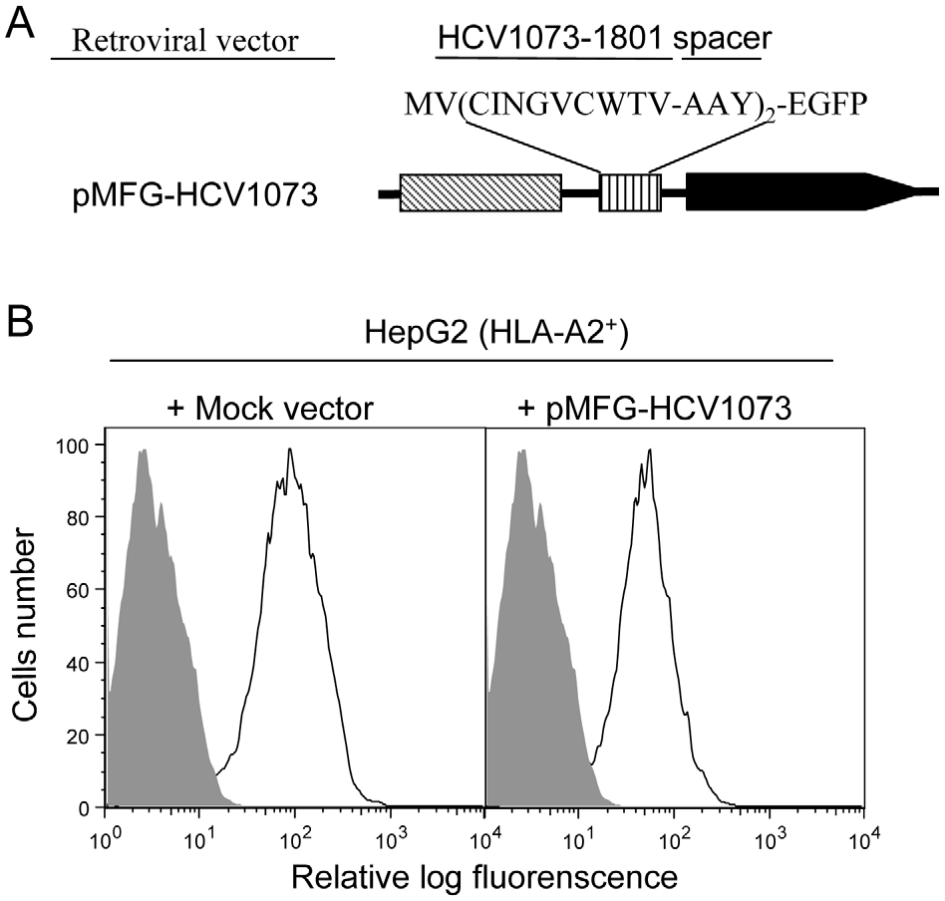
HCV NS3:1073–1081 expression in the hepatocellular carcinoma cell line HepG2. The HLA-A2^+^ HepG2 hepatocellular carcinoma cell line was transduced with the MFG retroviral vector encoding a fusion protein consisting of a the HCV NS3:1073–1081 peptide sequence fused in-frame with EGFP. (A) HCV NS3 1073–1801 minigene coding sequence fused to EGFP was inserted into a retrovirus vector pMFG. (B) HepG2 cells were transduced with pMFG EGFP or pMFG-HCV1073/EGFP. Transduced cells were enriched for uniform high antigen expression by FACS sorting based on EGFP expression. Open curves in each histogram represent transduced HepG2 cells (mock or HCV 1073 vevtor) while shaded curves represent untransduced cells. Each histogram represents the log green fluorescence of 1×10^5^ live cells as measured by flow cytometry.

The critical feature for a TCR gene-modified T cell is its ability to recognize endogenous antigen on the target-cell surface. However, human liver or HCC cells infected with HCV were not available for our experiments. Therefore, we established an HCV expressing HCC cell line to test the ability of our TCR-transduced T cells to recognize HCV^+^ liver tumor cells. An HCV expression construct was prepared by fusing the HCV NS3:1073–1081 minigene to the EGFP gene which was used as a marker to monitor the level of antigen expression by the HCC cells. The HCV/EGFP fusion construct was inserted into the retroviral vector pMFG (Figure 3A) which was used to transduce the HLA-A2^+^ hepatocellular carcinoma cell line, HepG2 (HLA-A2 expression is shown in Figure S1). HCV minigene-positive cells were defined by their EGFP expression as measured by flow cytometry (Figure 3B) and EGFP expressing cells were sorted by FACS for high and homogeneous antigen expression. The HCV^+^ HCC cells were then used as stimulators in cytokine release assays to evaluate the function of HCV TCR transduced T cells.

### HCV NS3:1073–1081 Antigen Recognition by HCV TCR-Transduced Jurkat Cells

**Figure 4:**
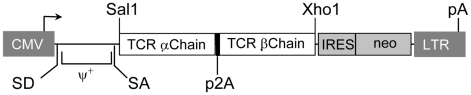
Structure of retroviral vectors used for TCR gene transfer and TCR expression on transduced cells. A modified SAMEN retroviral backbone was used for transferring TCR genes to alternate effectors. The retroviral vector contains the HCV TCR 1073 α and β chain genes fused by a 2A self cleavage peptide, under control of the hybrid MMLV/CMV promoter in the 5′ LTR. An IRES/neo cassette was included for G418 selection of transduced cells.

**Figure 5:**
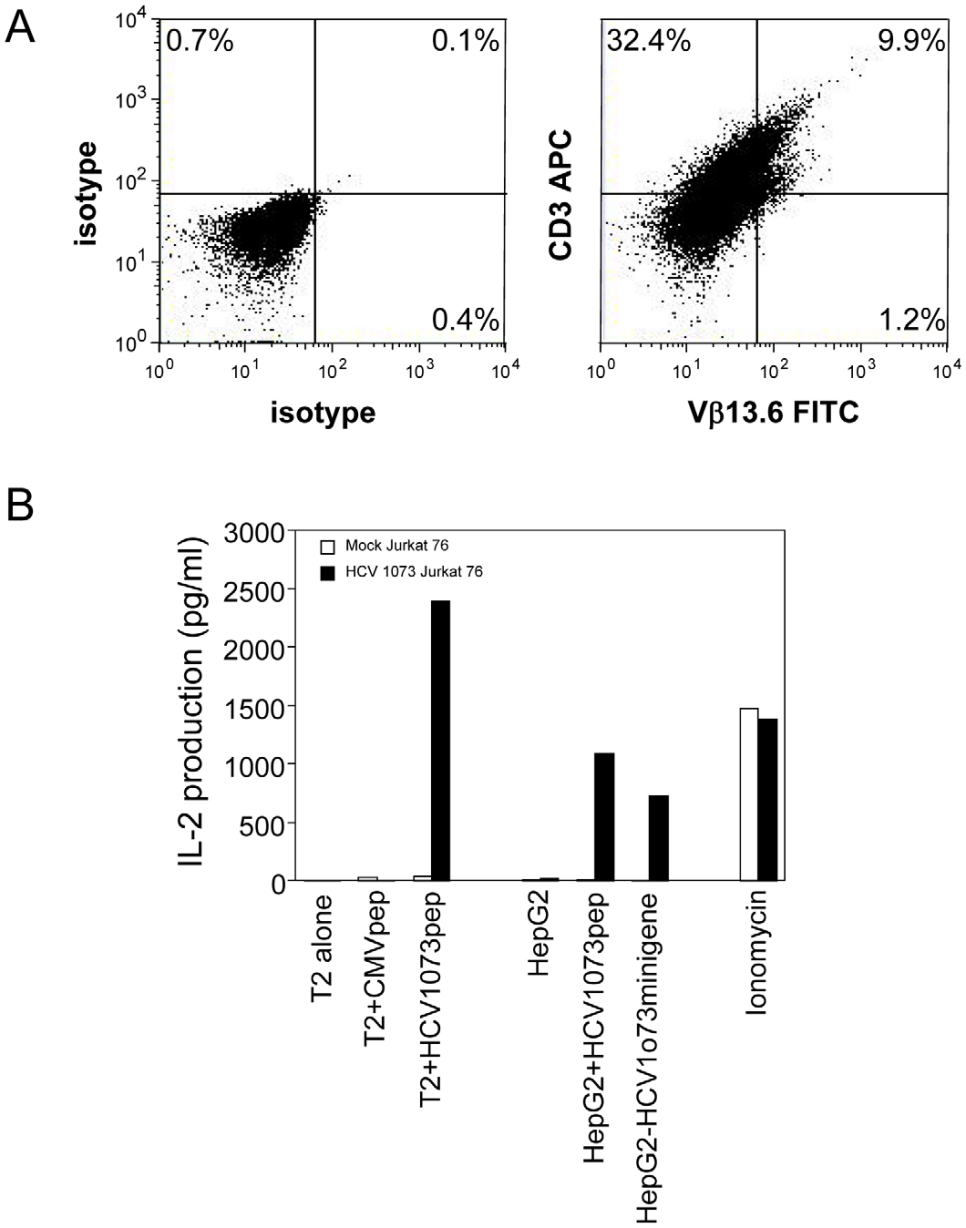
Expression and Function of the HCV TCR in Jurkat 76 cells. Jurkat 76 cells were transduced with the HCV TCR retroviral vector. (A) Expression of the HCV TCR in transduced Jurkat 76 cells was measured by CD3 and Vβ13.3 expression. Jurkat 76 cells were stained with anti-CD3 and anti-Vβ13.6 antibodies. Left panel: isotype control staining; right panel: anti-CD3 and anti-Vβ13.6 mAb staining. (B) Antigen recognition by HCV 1073 TCR transduced Jurkat cells was measured using IL-2 release assays. T2 or HepG2 cells were pulsed for 2 hr with 5 µg/ml of HCV NS3 1073–1081 peptide or CMVpp65 peptide. Peptide loaded cells or HCV^+^ HepG2 cells were incubated for 20 hr in microwells with the HCV TCR 1073 tranduced Jurkat 76 cells. The IL-2 production was measured by ELISA.

We have generated the recombinant retroviral construct containing the TCR α and β chains of the HCV NS3:1073–1081 reactive T cell clone (Figure 4). To verify the expression and function of this cloned TCR, we used this retroviral vector to transduce Jurkat 76 cells. Jurkat 76 cells are a TCR α^−^ and β^−^ derivative of the CD8^−^ human T cell lymphoma Jurkat cell line. Since Jurkat 76 cells are TCR negative, any introduced TCR would not have to compete with the endogenous TCR and its expression can be monitored by staining with anti-CD3 mAb. Furthermore, Jurkat 76 cells expressing a cloned TCR secrete IL-2 upon antigen stimulation in an antigen-specific fashion. Therefore, Jurkat 76 cells are an excellent model to evaluate the expression and function of any cloned TCR. As shown in Figure 5A, Jurkat 76 cells expressing the HCV NS3:1073–1081 TCR stained with anti-Vβ13.6 and anti-CD3 mAb's indicating the TCR could assemble on the surface of the Jurkat 76 cells. When stimulated with antigen, these HCV TCR transduced Jurkat 76 cells secreted significant IL-2 in response to T2 cells loaded with the HCV NS3:1073–1081 peptide but not T2 cells alone or T2 cells loaded with the CMV pp65:495–503 peptide (Figure 5B). The HCV TCR transduced Jurkat 76 cells also recognized HepG2 cells loaded with the HCV NS3:1073–1081 peptide or transfected to express the HCV NS3:1073–1081 epitope. It should be noted that despite the mock transfectants having higher expression of EGFP (Figure 3B), they were not recognized by the Jurkat cells expressing the HCV TCR. More importantly, recognition of HCV^+^ HepG2 cells by CD8^−^ Jurkat cells indicates our HCV TCR transfers CD8 independent tumor cell recognition to alternate effectors. These results indicate the HCV TCR is functional and has high affinity for antigen.

### Recognition of HCV^+^ Hepatocellular Carcinoma Cells by the HCV TCR-Transduced T Cells

**Figure 6:**
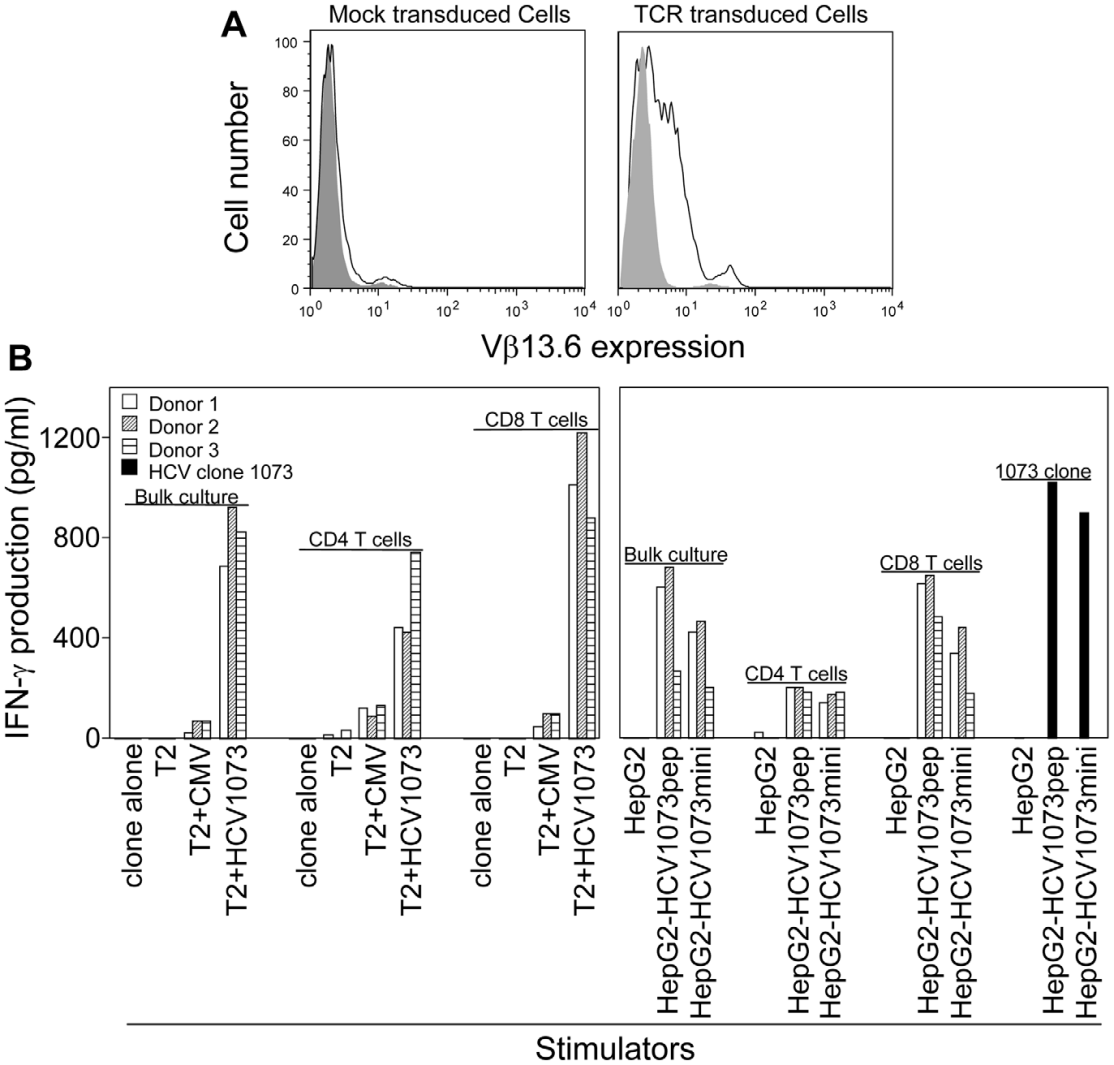
Antigen recognition by HCV TCR 1073-transduced PBLs. (A) Expression of HCV 1073 TCR on transduced PBLs. PBLs from three representative normal donors (Donors 1–3) were transduced with the HCV 1073 TCR retroviral vector. TCR-transduced cells and mock transduced T cells were stained with anti-Vβ13.6mAb. Solid gray: isotype control; Open white: anti-Vβ13.6mAb; left panel: mock vector transduced cells, right panel: TCR transduced cells. (B) Antigen recognition by HCV TCR 1073-transduced T cells. T2 cells or HepG2 cells were pulsed for 2 hr with 5 µg/ml of HCV NS3 1073–1081 peptide or CMVpp65 peptide. Peptide-pulsed T2 cells, HepG2 cells and HCV^+^ HepG2 cells were incubated for 20 hr in microwells with the HCV TCR 1073 T cells. The interferon-γ production was measured by ELISA.

Although Jurkat 76 cells are a good model cell line for verifying the function of a cloned TCR, they can't be used in preclinical animal studies or clinical trials to control HCV infections or the growth of HCV^+^ HCC cells. Therefore, it is critical to evaluate the function of normal PBL-derived T cells expressing our HCV TCR, particularly with regards to their ability to recognize a physiologically relevant target such as hepatocellular carcinoma cells. To accomplish this goal, we generated populations of HCV TCR transduced PBL-derived T cells from a total of seven normal healthy donors. The level of expression and the percent HCV TCR transduced T cells was measured by anti-Vβ13.6 mAb staining. The results from a typical HCV TCR transduced T cell culture is shown in Figure 6A. Compared to the isotype control, mock transduced T cells contained 1.1% Vβ13.6 staining cells. This represents the frequency of Vβ13.6 staining cells present in normal PBL. The HCV TCR transduced T cell cultures contain 36% Vβ13.6 staining cells with the level of TCR expression being variable as expected by a TCR-transduced T cell population. These results indicate that our HCV TCR can be efficiently expressed by PBL-derived T cells from normal donors. However, despite having anywhere from 20%–40% Vβ13 expressing T cells in the HCV TCR transduced T cell cultures, only about 0.25% of the CD4^+^ and CD8^+^ T cells bind HCV 1073 peptide loaded pentamers (Figure S2). We and others have found that tetramer binding does not always correlate with TCR expression and function so this result was not surprising [38]–[40].

**Figure 7:**
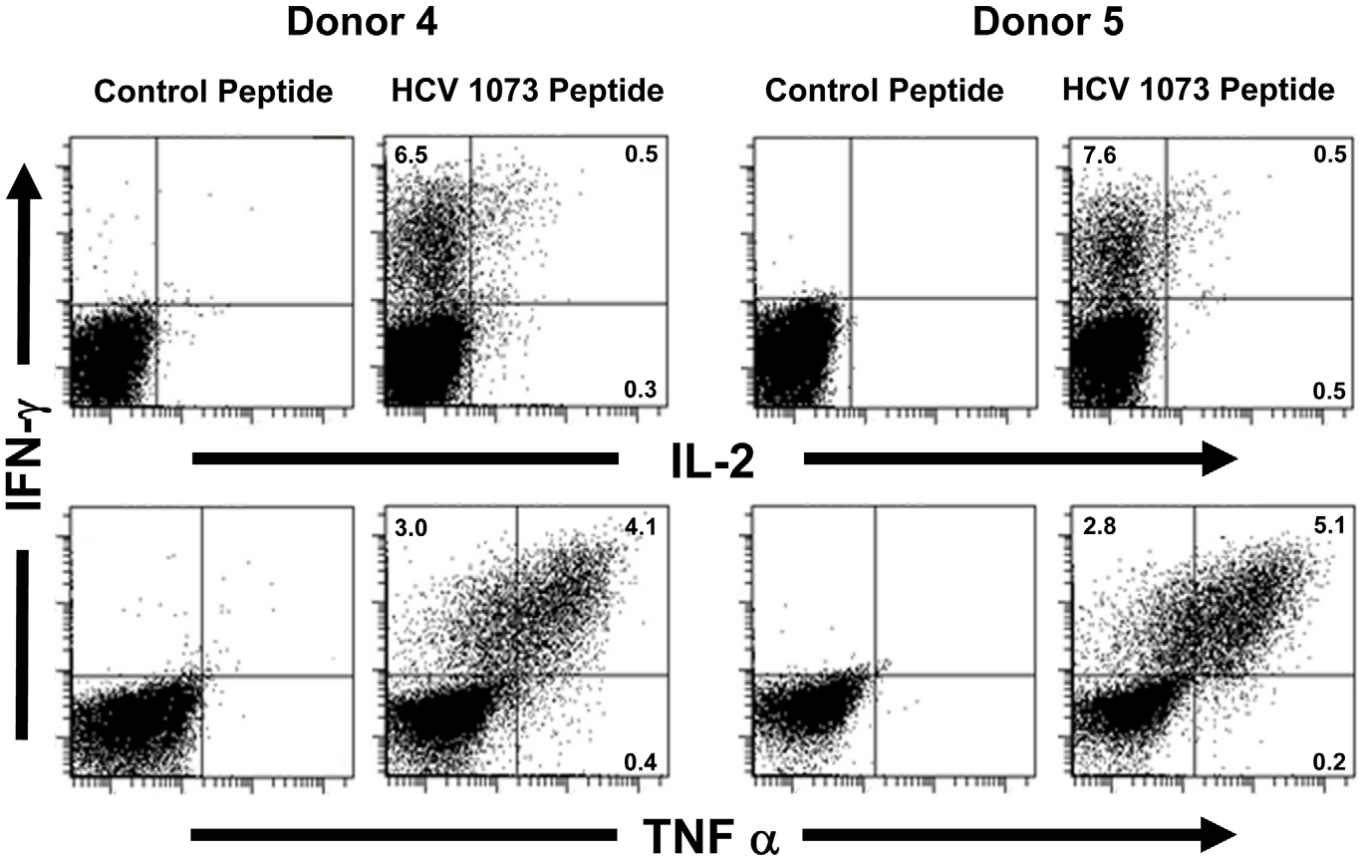
Intracellular cytokine staining of HCV TCR transduced T cells. Two representative HCV TCR transduced T cell cultures (Donors 4 and 5) were stimulated with T2 cells loaded with the cognate HCV 1073 peptide or the control HCV 132 peptide and stained for intracellular IFN-γ (Y-axis), IL-2 (X-axis, top row), and TNF-α (X-axis, bottom row). The amount of fluorescence was measured by flow cytometry and each histogram represent 10^4^ cells.

**Figure 8:**
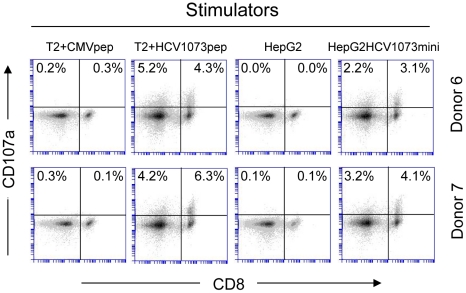
CD107a staining of HCV TCR 1073-transduced T cells. HCV TCR1073 transduced T cells were stimulated for 20 hours with peptide loaded T2 cells or HepG2 tumor cells. T2 cells were pulsed for 2 hr with 5 µg/ml of the HCV NS3 1073–1081 peptide or the CMV pp65 control peptide. Parental HepG2 tumor cells or HCV^+^ HepG2 cells were used as stimulators. Following the stimulation, the T cells were collected and stained with anti-CD107a mAb. Cells were gated on CD3^+^ fraction and the numbers in the quadrants represent the percentage of either CD4^+^ or CD8^+^ cells staining for CD107a expression. The amount of fluorescence was measured by flow cytometry and each histogram represent 10^4^ cells.

PBL-derived T cells from the normal donors were transduced with our HCV TCR and assessed for their ability to recognize antigen. Using a combination of cytokine release and intracellular cytokine staining, we evaluated the antigen reactivity of each of the HCV TCR transduced T cell cultures. All of the bulk T cell cultures produced significant amounts of interferon-γ when stimulated with T2 cells loaded with the HCV NS3:1073–1081 peptide but not T2 cells alone or T2 cells loaded with the control CMV pp65:495–503 peptide (Figure 6B, Figure 7, and Figure S3). These HCV TCR transduced T cells did produce TNF-α and IL-2 upon stimulation with peptide loaded T2 cells (Figure 7 and Figure S3). The HCV TCR transduced bulk T cells also efficiently recognized HepG2 cells (which naturally express HLA-A2; Figure S1) loaded with HCV NS3:1073–1081 peptide or transfected to express the HCV NS3:1073–1081 epitope (Figure 6 and Figure S3). It should be noted that despite the mock transfectants having higher expression of EGFP (Figure 3B), they were not recognized by the normal PBL-derived T cells expressing the HCV TCR. Moreover, TCR-transduced PBL demonstrated cytotoxicity as shown by the production of CD107a (Figure 8). Therefore, our HCV TCR efficiently engineers normal PBL-derived T cells to recognize HCV peptide loaded targets with a polyfunctional response (production of IFN-γ, IL-2, TNF-α and CD107a).

Recognition of peptide loaded targets by HCV TCR-transduced T cells confirms the reactivity of our HCV TCR in normal PBL-derived T cells. However, it is more important to verify the recognition of antigen presented by HCV^+^ cells such as HCC cells. The recognition of HCV^+^ HepG2 cells by the HCV TCR transduced Jurkat 76 cells indicated that our HCV TCR transfers CD8-independent tumor cell recognition to alternate effectors (Figure 5B). We have previously reported that CD8 independent TCR's are capable of generating MHC class I restricted CD4^+^ T cells making it possible to provide patients with a novel source of T cell help [41], [42]. To determine if our HCV TCR can generate MHC class I restricted CD8^+^ effector and CD4^+^ helper T cells, we transduced PBLs derived T cells from three healthy donors and purified the CD4^+^ and CD8^+^ T cells to greater than 99% purity using immunomagnetic beads to measure cytokine production by ELISA (Figure 6 and Figure S3) or analyzed each subset for intracellular cytokine production or CD107a expression (Figure 7 and Figure 8). CD4^+^ and CD8^+^ T cells, transduced to express our HCV TCR, produced significant amounts of interferon-γ, TNF-a, IL-2, and CD107a when stimulated with HCV peptide loaded T2 cells or HepG2 cells but not controls. Importantly, the HCV TCR-transduced CD4^+^ T cells secreted significant amounts of cytokine when stimulated with HCV^+^ tumor cells. These results indicate that our HCV TCR can engineer both CD8^+^ T cells and CD4^+^ T cells to recognize HCV^+^ cells. Also, our ability to generate MHC class I restricted CD4^+^ T cells raises the possibility that we can provide any HCV patient with a source of autologous HCV-reactive T helper cells which has been implicated in eradicating HCV infections [43].

### Relative Functional Avidity of HCV TCR-Transduced T Cells

**Figure 9:**
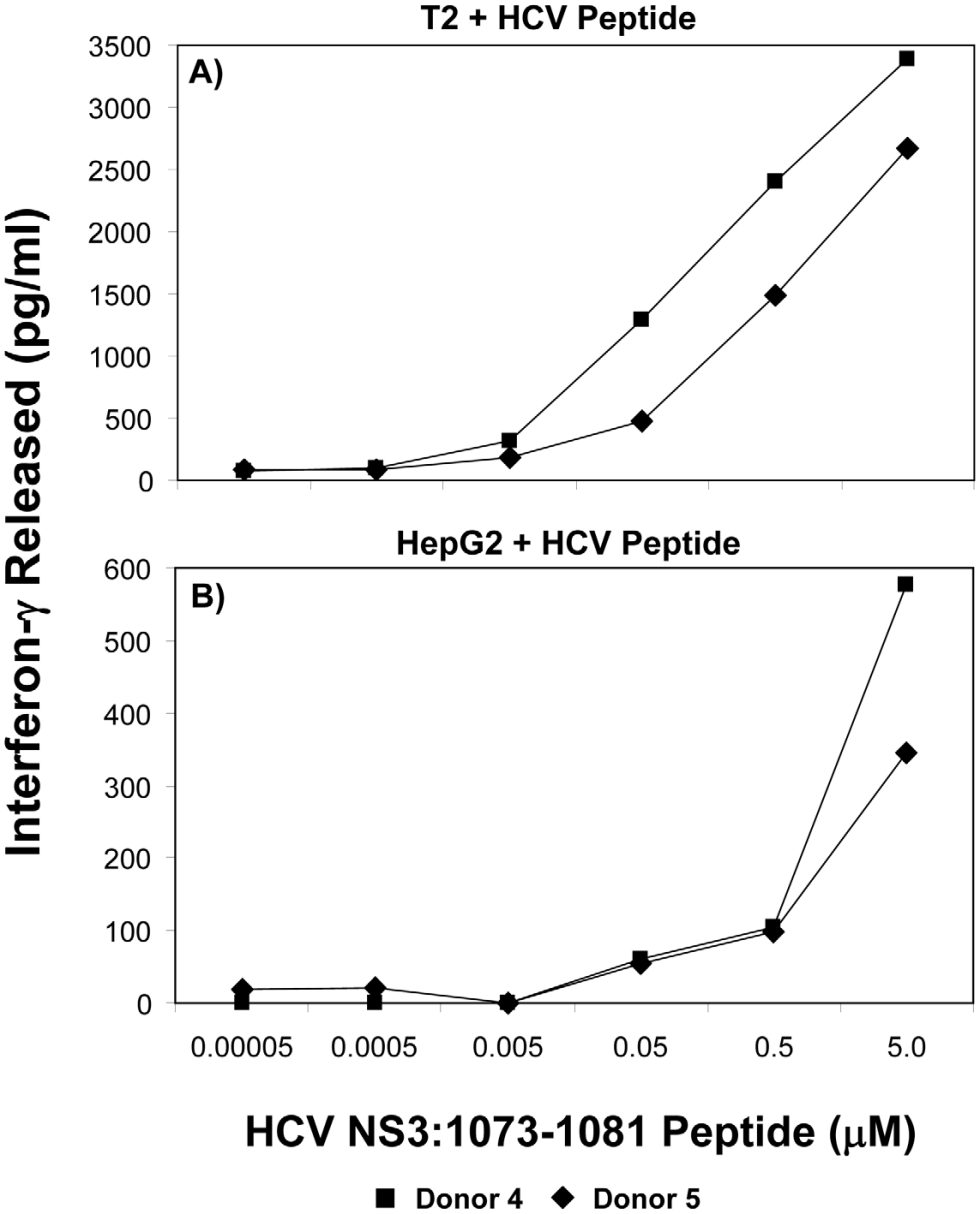
Functional avidity of HCV TCR 1073-transduced T cells. The functional avidity of HCV TCR transduced PBL-derived T cell cultures was measured interferon-γ release assays. T2 cells (Panel A) or HepG2 cells (Panel B) were loaded for 2 hr with varied concentrations of HCV NS3 1073–1081 peptide (0.05–5,000 nM). These peptide-loaded T2 or HepG2 cells were cocultured for 20 hr in microwells with two representative HCV TCR transduced T cell cultures (Donors 4 and 5) and the amount of interferon-γ produced was measured by ELISA. Background levels of interferon-γ produced when the T cells were cocultured with T2 cells alone or HepG2 cells were 38/0 (Donor 4) and 58/6 (Donor 5). The amount of interferon-γ produced when the T cells were cocultured with HepG2 cells expressing the HCV 1073 minigene was 717 and 418 (Donors 4 and 5 respectively).

It has been shown that there is a correlation between the functional avidity of a T cell and its ability to recognize tumor cells or virus-infected cells [43]–[47]. Furthermore, T cells expressing a high affinity TCR have been shown to be exquisitely sensitive to low levels of antigen [48]. T cells with identical specificities, but different functional avidities, influence each other during activation and homeostatic proliferation [49]. T cells exhibiting increased sensitivity to stimulation, or a lower threshold, are said to have a relatively high functional avidity [44]–[47]. T-cell responsiveness to peptide is commonly used as a measure of T cell avidity as it provides a measure of the stimulation threshold required to activate T cell effector functions. Relative avidities were evaluated by measuring T-cell interferon-γ production. To test the avidity of the HCV TCR-transduced T cells, we loaded different peptide concentrations on T2 or HepG2 cells and incubated with HCV TCR transduced T cells. The ability of the transduced T cells to produce interferon-γ was measured under conditions of increasing concentrations of peptide stimulation. As shown in Figure 9, the two representative HCV TCR transduced T cell cultures (Donors 4 and 5) had high avidity for antigen since they secreted significant amounts of interferon-γ when stimulated T2 cells loaded with 5 nM or less of peptide. Similar results were found with three other HCV TCR transduced T cells cultures (Donors 1–3) with the functional avidity of the HCV TCR transduced T cells being approximately half a log lower than the parent T cell clone (<1.0 vs <0.5 nM) (Figure S4).

In contrast, Peptide loaded HepG2 cells were not recognized as well as the T2 cells since it required between 50–500 nM peptide to stimulate the HCV TCR transduced T cells. This was not surprising since our HepG2 cells but not our T2 cells have their MHC class I molecules loaded with peptides requiring peptide exchange on the HepG2 cells for T cell recognition to occur. In fact, the HCV TCR transduced T cells did not recognize the HepG2 cells loaded with 5 µM peptide as well as the HepG2 cells expressing the HCV 1073 minigene (576/345 pg/ml vs 717/418 pg/ml respectively for donors 1 and 2) further supporting the notion that exogenous peptide loading was less efficient than endogenous peptide loading. Compared to other published studies, our functional avidity measurements using peptide loaded T2 cells indicate our HCV TCR transduced T cells have relatively high avidity for antigen.

## Discussion

The relationship between T cell avidity and the clearance of viral infections and tumor cells has been well documented [44], [45]. Many studies have been directed at elucidating the relationship between T-cell activity and TCR affinity, dissociation rate, and CD8^+^ dependence. CD8^+^ played an essential role in T-cell recognition of low-affinity T-cell reactions [48]. However, we speculated that any TCR that can bind peptide/MHC complexes without CD8 would have higher relative affinity than a TCR that requires CD8^+^ for binding. To date, only a limited number of CD8-independent TCRs have been cloned and characterized [50]. The novel HCV TCR described herein exhibits CD8-independent target cell-recognition since the HCV TCR-transduced CD4^+^ T cells could secret interferon-γ and IL-2 when stimulated with peptide-loaded targets or HCV^+^ HCC cells (Figure 6B and Figure S3). Based on this study, we conclude that the affinity of this HCV TCR is higher than other TCRs that require the CD8 coreceptor for target-cell recognition, These results indicate that T cells isolated from patients with chronic HCV infection can have high affinity TCRs and these TCRs may be important for developing novel TCR-based gene therapy studies. Thus, we have identified another high affinity TCR that could be used to engineer normal PBL-derived T cells for clinical application.

CD4^+^ T cells are thought to contribute to anti-viral immune responses by secreting cytokines, thereby providing help to CD8^+^ T cells [51], [52]. Antigens are taken up by antigen presenting cells which may activate CD4^+^ T cells to secrete either Th1 or Th2 cytokines. By producing Th1-cytokines like interferon-γ and IL-2 CD4^+^ T cells contribute to anti-viral immune responses providing help to CD8^+^ T and B cells. Furthermore, CD4^+^ T cell lines and clones can display direct cytotoxic effector function [51], [52]. The identified HCV TCR has been successfully transduced into CD4^+^ T cells and these TCR-transduced CD4^+^ T cells may not only provide help to CD8^+^ T cells, but also directly act on the HCV^+^ target cells such as HCV-infected cells and HCV^+^ HCC cells. This is especially important for clearing HCV infection because one of the fundamental problems typical of chronic HCV infection is a weak or absent HCV- specific CD4^+^ T-cell response [43], [53].

The instability of the HCV genome makes the identification of this high affinity HCV NS3:1073–1081 TCR particularly important. We and others have previously shown that T cells can express two functional TCRs capable of recognizing both target antigens [54]–[56]. Therefore, when combined with our previously identified HCV NS3:1406–1415 TCR, T cells expressing both TCRs might be effective against HCV immune escape variants for treatment of HCV-associated diseases. Adoptive transfer of HCV TCR-transduced T cells may show promise as a new treatment for patients with chronic HCV infection or HCV-related malignancies, particularly in light of the recent demonstration that HCC express HCV antigens [57].

## Materials and Methods

### Cell Lines, Media, and Reagents

T2 and HepG2 cells were obtained from the American Type Culture Collection (Rockford, MD). The TCR-negative Jurkat 76 cell line has been described elsewhere [58]. Unless otherwise indicates, All medium components were obtained from Mediatech (Herndon, VA) unless otherwise noted. Jurkat 76 and T2 cell lines were maintained in complete medium consisting of RPMI 1640 medium supplemented with 10% fetal bovine serum (Tissue Culture Biologicals, CA) 100 U/mL penicillin, 100 µg/mL streptomycin. Plat-A cells [59], [60] and HepG2 cells were maintained in Eagle's medium supplemented as described above. TCR-transduced Jurkat 76 cells were maintained in RPMI medium as described above supplemented with 2 mg/mL G418.

To engineer tumor cell lines to express HCV sequences, we first inserted synthetic oligonucleotides encoding the HCV NS3:1073–1031 epitope into the retroviral vector, pMFG-EGFP. Briefly, oligonucleotides encoding the HCV NS3:1073–1031 epitope and containing mutated Nco I restriction sites (forward: 5′-catgTGCATCAATGGGGTATGCTGGACTGTCgctgcttatgg-3′; reverse 5′- ccataagcagcGACAGTCCAGCATACCCCATTGATGCAcatg-3′) were synthesized and annealed. The underlined base pairs indicate the overhang for the ligation of the double stranded oligonucleotides into the pMFG-EGFP vector using a shotgun ligation strategy as described [41]. The recombinant vector was transiently transfected into Plat-A packaging cells and the retrovirus containing supernatant was collected for transduction of HepG2 cells. The expression of the HCV NS3:1073–1081 minigine in the transduced HepG2 cells was confirmed based on the EGFP expression as measured by flow cytometry. The EGFP positive cells were sorted for high and uniform expression and the resulting HCV^+^ HCC cell line was established.

### T Cells

All T cells were maintained in AIM V medium (Invitrogen, GIBCO) supplemented with 5% heat-inactivated pooled human AB serum (Valley Biomedical, Inc), 100 U/mL penicillin, 100 µg/mL streptomycin and 300 IU/mL recombinant human IL-2 (rhIL-2; Novartis Pharmaceuticals Corporation, East Hanover, NJ) at 37°C in a humidified 5% CO_2_ incubator. The isolation and characterization of HCV-reactive T-cell clones has been previously described [32]. The HCV NS3:1073–1081-reactive CD8^+^ T-cell clone used in this study was isolated from a patient with a chronic HCV infection.. All PBMC used in this study came from apheresis products purchased from (Research Blood Components, L.L.C., MA). Normal PBL-derived T cells were isolated from the PBMC cells of three independent normal healthy donors using Ficoll-Hypaque density gradient centrifugation. The HCV T cell clone and the TCR-transduced T cells were expanded using 30 ng/mL anti-CD3 monoclonal antibody (Ortho Biotech, Raritan, NJ) and 300 IU/mL rhIL-2 in the presence of irradiated pooled allogeneic peripheral blood mononuclear cells as feeders as previously described [61].

### Peptides

HCV NS3:1073–1081 (CINGVCWTV), HCV NS3:1406–1415 (KLVALGINAV), CMV pp65:495–503 (NLVPMVATV) were obtained from Synthetic Biomolecules (San Diego, CA). T2 or HepG2 cells were loaded with each peptide by incubating 1×10^6^ cells/ml in complete medium containing 5 µg/ml (unless otherwise noted) of peptide at 37°c for 2 hours. Peptide-loaded cells were washed with fresh complete medium before coculture with responders.

### TCR α and β Chain Identification

The TCR α chain from the HCV NS3-1073–1081-reactive T-cell clone was identified by 5′ RACE as previously described [37], [60]. Briefly, total RNA was isolated from 2.5×10^6^ cells using TRIzol (Invitrogen), first-strand cDNA was synthesized, and the TCR cDNAs were amplified using the SMART RACE cDNA Amplification kit (Clontech Laboratories, Inc, Mountain View, CA). Fragments containing random TCR α chains were amplified using the Advantage 2 PCR Enzyme system (Clontech Laboratories, Inc) using the universal primer A mix and a TCR α constant region (AC) specific reverse primer. The random PCR products were ligated into TA PCR2.1-Topo cloning vector, and transformed into Escherichia coli TOP 10 competent cells (Invitrogen). Bacterial clones were screened for the presence of TCR α chain cDNA by PCR and random 5′ RACE clones were sequenced using fluorescent dye labeled ddNTPs (Applied Biosystems Inc, Foster City, CA). DNA sequence analysis revealed a single productively rearranged TCR α chain which used the AV20s1. The full-length α chain was amplified from cDNA using an AV20s1 forward (5-AAGTCGACGTTTGCACCTAGAATATGAGGCAAGTGGCG-3) and an AC reverse (5-AAGTCGACTCAGCTGGACCACAGCCGCAG-3) primer containing Sal I restriction sites for subsequent subcloning. The PCR product was ligated into the pCR 2.1 TA cloning vector (Invitrogen), and transformed into Escherichia coli TOP 10 competent cells (Invitrogen). Bacterial clones were screened for the presence of the α chain cDNA via PCR and were sequenced to ensure that no errors had occurred during PCR amplification.

The TCR β chain from the HCV-reactive T-cell clone was identified via RT-PCR using a panel of TCR β chain V region (BV) subfamily specific primers as previously described [37]. Briefly, total RNA was isolated and first strand cDNA was prepared from 2.5×10^6^ T cells using the procedure as described above for the α chain identification. A single band was amplified using the BV13 subfamily specific primer and the TCR β chain was identified as BV13s6 based on known TCR BV genomic DNA sequences. The full-length β chain was amplified from cDNA using a BV13s6 forward (5′-CTCGAGGCACCTGCCATGAGCATCAGCCTC-3′) and a BC2 reverse (5′-AACTCGAGCTAGCCTCTGGAATCCTTTCTCTTGACCAT-3′) primer that each contained Xho I restriction sites for subsequent subcloning. The PCR fragment was ligated into the pCR 2.1 TA cloning vector, and transformed into Escherichia coli TOP 10 competent cells. Bacterial clones were screened for the presence of the β chain gene, and recombinant clones were sequenced to ensure that no errors had occurred during PCR amplification.

### Retroviral Vector Construction

The SAMEN CMV/SRα retroviral vector has been previously described [14] and was used as the backbone for all retroviral constructs. The TCR α and β chains were linked by a 2A self cleavage peptide. The HCV TCR α chain, 2A linker and β chain fusion gene fragment was inserted into the Xho I and Sal I restriction sites of the retrovirus vector. The configuration of the retroviral vector used in this study is shown in Figure 4.

### Retroviral Transduction

Retroviral supernatants were prepared using a transient transfection protocol as described [41]. Briefly, 5×10^6^ Plat-A cells were plated in 10 cm poly-D-Lysine coated plates in 10 ml DMEM containing 10% FBS without antibiotics at sufficient density to provide 60% to 70% confluence after 24 hr. Cells were transiently cotransfected with 9 µg of retroviral vector DNA and 4.5 µg of plasmid DNA containing the vesicular stomatitis virus envelope gene using Lipofectamine 2000 (Invitrogen). Transfection medium was replaced with 10 ml complete medium after 6 h incubation, and retroviral supernatants were collected after 48 hr.

Jurkat 76 were transduced by spinoculation as described [14]. Briefly, Jurkat 76 cells were resuspended at a concentration of 2×10^6^/ml in retroviral supernatant containing 8 µg/mL polybrene. 1 ml of cells was added to each well of a 24-well flat-bottom tissue culture plate then spun for 90 min at 1000×g at 32°C. After centrifugation, the cells were resuspended in their wells, incubated for 4 hr at 37°C, and 1 mL fresh complete medium was added to each well. This spinoculation procedure was repeated the next day using fresh retroviral supernatant. After 24 hr, the transduced cells were resuspended at 5×10^5^/ml in culture medium and transduced cells were selected by the addition of 2 mg/mL of G418.

T cells were transduced by spinoculation as described [14]. Briefly, retrovirus was first loaded onto RetroNectin-coated 24-well flat-bottom non-treated tissue culture plates by adding 1 mL of fresh retroviral supernatant per well and the plates were spun for 2 hr at 2,000×g at 32°C. T cells derived from healthy donors were activated using 50 ng/mL anti-CD3 monoclonal antibody and 300 IU/mL rhIL-2. The activated T cells were resuspended at 1×10^6^ cells/mL with culture medium supplemented with 300 IU/mL of rhIL2. The T cells were then gently added to the plates and mixed with the viral supernatnant. The plates were continuously centrifuged at 1,000×g for 10 min at 32°C. After 24 hours, the transduced T cells were selected by adding 1 mg/mL of G418. The CD4^+^ T cells were sorted from the TCR-transduced T cells by positive selection with magnetic beads. The purity of separated CD4^+^ and CD8^+^ cells was confirmed by FACS analysis.

### Immunofluorescence Staining

All T cell and tumor cell lines were stained for immunofluorescence with fluorochrome conjugated anti-CD3 (APC), Vβ13.6 (FITC), anti-CD4 (APC), anti-CD8 (FITC), anti-CD107a, (PE) and anti-HLA-A2 (PE) purchased from BD Biosciences, San Diego, CA. PE conjugated HCV NS3:1073–1081 or HCV Core:132–140 peptide loaded HLA-A2 pentamers were purchased from Proimmune Ltd., Oxford, United Kingdom. In all experiments, 10^6^ live cells were stained for 30 minutes on ice with individual monoclonal antibodies or pentamers. Cells were washed and stained with a second reagent or analyzed immediately on an Accuri C6 or BD FACSCalibur flow cytometer. The log fluorescence of a minimum of 10^6^ cells was analyzed for each sample.

### Cytokine Release Assays

Antigen reactivity by the HCV-reactive T cell clones and HCV TCR transduced cells was measured in cytokine release assays as described [13]. Briefly, 1×10^5^ responder and stimulator cells were cocultured in a 1∶1 ratio in 96-well U-bottom tissue culture plates in 200 µL complete medium. For the Jurkat 76 experiments, 10 ng/mL of PMA (Sigma-Aldrich, St. Louis, MO) was added to each well. As a positive control for Jurkat stimulation, maximal cytokine release was obtained by the addition of 1 µg/mL ionomycin (Sigma-Aldrich). Cocultures were incubated at 37°C for 20 hours, and then supernatants were harvested. The amount of cytokine released was measured via ELISA using monoclonal antibodies to interferon-γ (Pierce, Rockford, IL) or IL-2 (R&D Systems, Minneapolis, MN).

### Intracellular Cytokine Staining

Multiparameter flow cytometry was performed using a BD FACSCanto II instrument (BD Biosciences, San Jose, CA) and analyzed using FACSDiva software (BD). Antibodies for cell surface CD3, CD4 and CD8 and for intracellular IFN-γ, TNF-α, and IL-2 were purchased from BD or eBioscience (San Diego, CA). Transduced T cell cultures were stimulated for 6 hours at 37°C in the presence of brefeldin A (Sigma-Aldrich) with equal numbers of T2 cells that had previously been loaded with the TCR-specific antigen (HCV-1073 peptide, 1 µg/ml) or with another HCV-derived A2-restricted control peptide (HCV-132, 1 µg/ml). After stimulation cells were stained for surface antigens, fixed for 30 minutes at 4°C in 100µl Fix and Perm Medium A (Caltag, Burlingame, CA), permeabilized using 100µl Fix and Perm Medium B (Caltag) and incubated with anti-cytokine antibodies for 1 hour at 4°C. Cell suspensions were then washed in PBS-BSA-Azide and fixed in 200 µl 1% PFA and acquired after 1 hour.

### CD107a Expression Assay

CD107a expression was used as a surrogate marker to assess the cytolytic ability of HCV 1073 TCR transduced T cells. HCV 1073 TCR transduced T were cocultured with a panel of stimulators using methods similar our cytokine release assays described above. Stimulators included T2 cells loaded with the HCV NS3:1073–1081 or CMV pp65;495–503 and tumor targets (HepG2 and HepG2 expressing the HCV 1073 minigene). Briefly, 1×10^5^ responder and stimulator cells were cocultured in a 1∶1 ratio in 96-well U-bottom tissue culture plates in 200 µL complete medium. Cocultures were incubated at 37°C for 20 hours, and then cells were harvested and washed. The cells were stained with anti-CD3 mAb, anti-CD8 mAb and anti-CD107a mAb (BD Pharmingen, San Diego, CA) and were analyzed by flow cytometer. Each histogram represents the log fluorescence of 10^4^ live T cells (gated using CD3 staining).

## Supporting Information

Figure S1 HLA-A2 expression on human HCC lines. HepG2 HCC cells were used throughout this study as stimulator cells for the HCV TCR transduced T cells. To confirm their HLA-A2 expression levels, HepG2 cells were stained with PE conjugated anti-HLA-A2 mAb (solid curve) or and isotype control mAb (open curve) and the amount of fluorescence staining was quantified by flow cytometry. As staining controls, the HLA-A2 negative HCC cell line Huh-7 and an HLA-A2 transfectant was stained. Each histogram represents the log fluorescence of 10^4^ live cells.
(0.08 MB TIF)

Figure S2 Pentamer staining of HCV TCR transduced T cells. HCV TCR transduced normal PBL-derived T cells were stained HCV peptide loaded pentamers. Two representative TCR transduced T cell cultures (Donors 4 and 5) and a normal donor untransduced cells (Donor 5) were stained with anti-CD4 mAb, anti-CD8 mAb, and HLA-A2 pentamers loaded with the HCV NS3:1073–1081 peptide. The percent pentamer positive CD8^+^ T cells (upper row) and CD4^+^ T cells (lower row) is shown in each histogram. Each histogram represents the log fluorescence of 10^4^ live cells.
(0.23 MB TIF)

Figure S3 Cytokine production by HCV TCR 1073-transduced T cells. PBMC from two normal donors (donors 4 and 5) were transduced to express the HCV TCR 1073 and were assessed for cytokine secretion. T2 cells were pulsed for 2 hr with 5 µg/ml of the HCV NS3 1073–1081 peptide or the CMV pp65 control peptide. Peptide-pulsed T2 cells, HepG2 cells and HCV^+^ HepG2 cells were cocultured for 20 hr in microwells with the HCV TCR 1073 T cells. The production of IFN-γ and IL-2 were measured by ELISA.
(0.06 MB TIF)

Figure S4 Relative avidity of HCV TCR 1073-transduced T cells. The functional avidity of HCV TCR transduced PBL-derived normal T cells was compared to the parent HCV NS3:1073:1081 T cell clone using interferon-γ release assays. T2 cells were loaded for 2 hr with varied concentrations of HCV NS3 1073–1081 peptide (0.01–1000 nM). These peptide-loaded T2 cells were cocultured for 20 hr in microwells with three different HCV TCR transduced T cell cultures or the parent HCV 1073-reactive T cell clone. The amount of interferon-γ produced was measured by ELISA.
(0.06 MB TIF)
