# Supplementary figures and images for: Transduction of Human T Cells with a Novel T-Cell Receptor Confers Anti-HCV Reactivity
Source: PLoS Pathog. 2010 Jul 29;6(7):e1001018. doi: 10.1371/journal.ppat.1001018 (PMC2912399; doi:10.1371/journal.ppat.1001018)

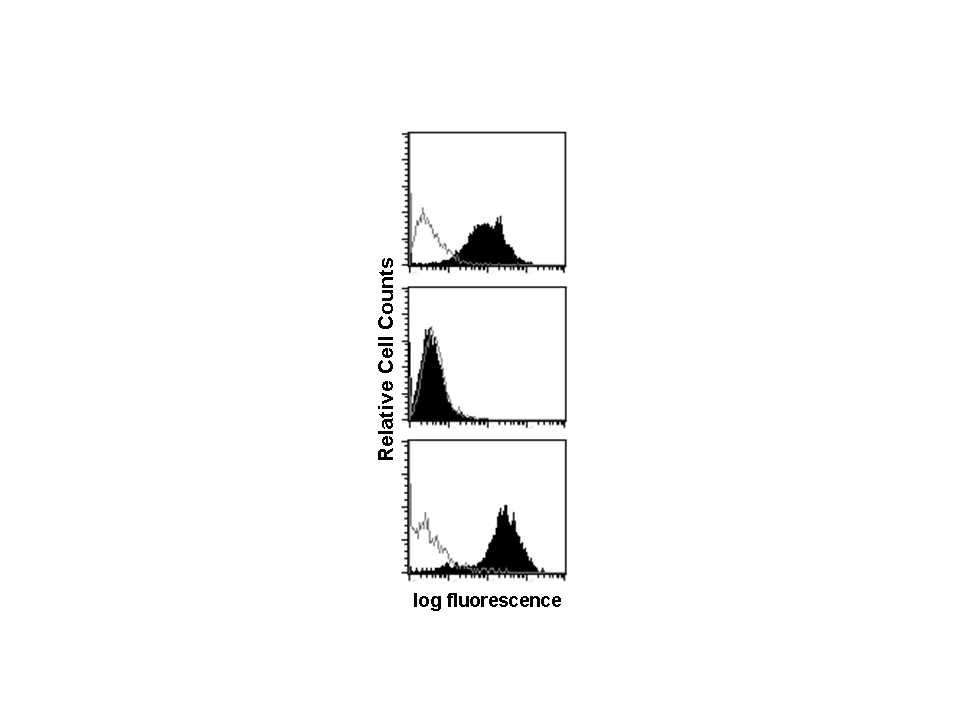

Supplement: Figure S1 — HLA-A2 expression on human HCC lines. HepG2 HCC cells were used throughout this study as stimulator cells for the HCV TCR transduced T cells. To confirm their HLA-A2 expression levels, HepG2 cells were stained with PE conjugated anti-HLA-A2 mAb (solid curve) or and isotype control mAb (open curve) and the amount of fluorescence staining was quantified by flow cytometry. As staining controls, the HLA-A2 negative HCC cell line Huh-7 and an HLA-A2 transfectant was stained. Each histogram represents the log fluorescence of 104 live cells. (0.08 MB TIF) [file ppat.1001018.s001.tif]

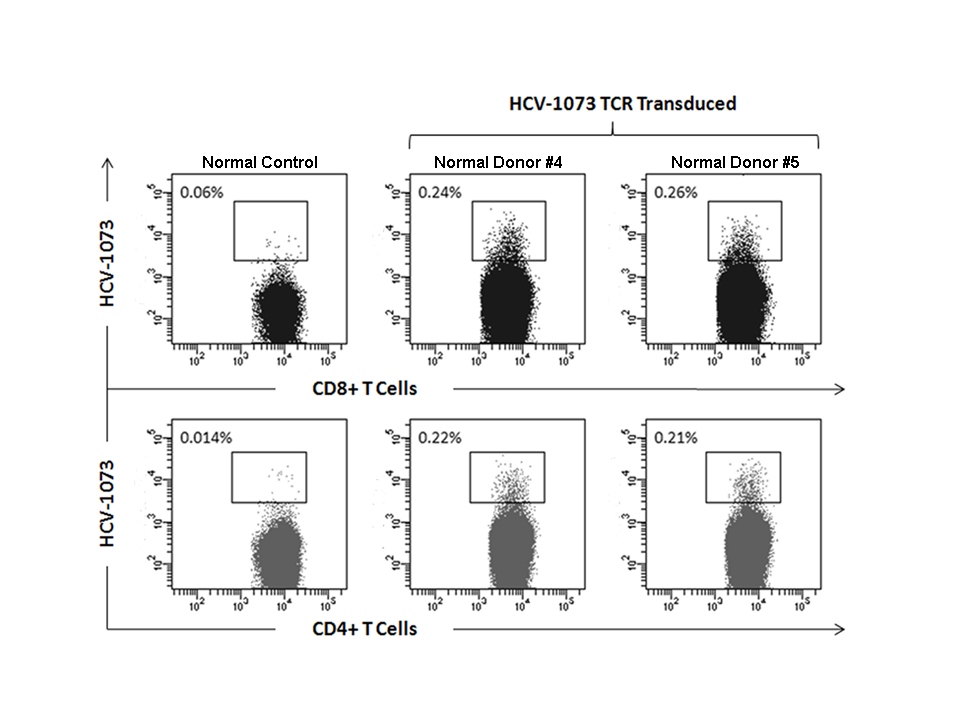

Supplement: Figure S2 — Pentamer staining of HCV TCR transduced T cells. HCV TCR transduced normal PBL-derived T cells were stained HCV peptide loaded pentamers. Two representative TCR transduced T cell cultures (Donors 4 and 5) and a normal donor untransduced cells (Donor 5) were stained with anti-CD4 mAb, anti-CD8 mAb, and HLA-A2 pentamers loaded with the HCV NS3:1073–1081 peptide. The percent pentamer positive CD8+ T cells (upper row) and CD4+ T cells (lower row) is shown in each histogram. Each histogram represents the log fluorescence of 104 live cells. (0.23 MB TIF) [file ppat.1001018.s002.tif]

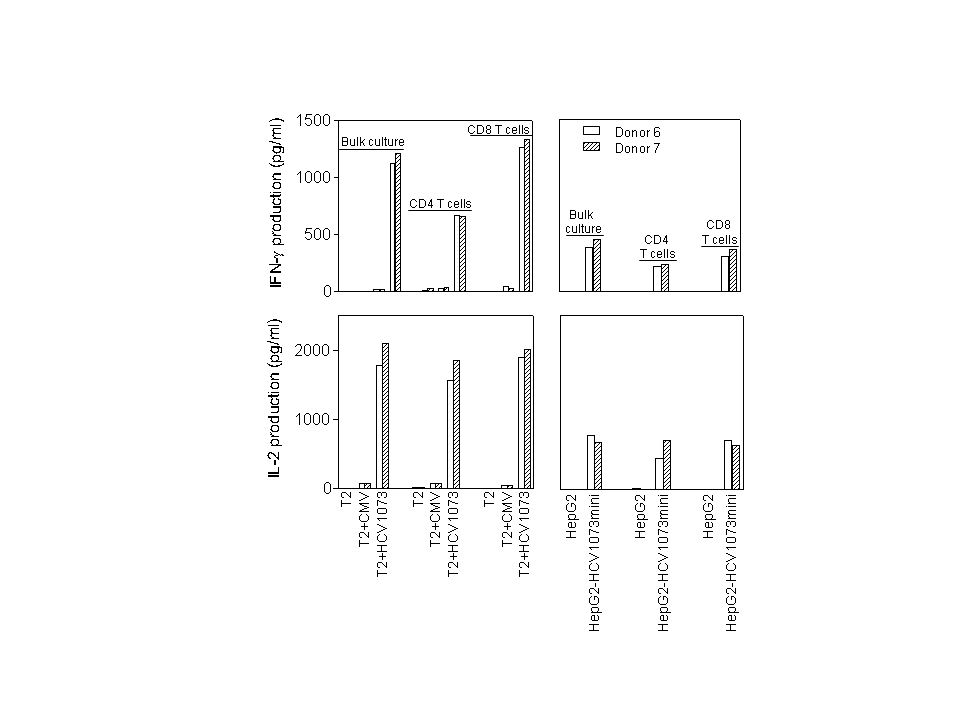

Supplement: Figure S3 — Cytokine production by HCV TCR 1073-transduced T cells. PBMC from two normal donors (donors 4 and 5) were transduced to express the HCV TCR 1073 and were assessed for cytokine secretion. T2 cells were pulsed for 2 hr with 5 µg/ml of the HCV NS3 1073–1081 peptide or the CMV pp65 control peptide. Peptide-pulsed T2 cells, HepG2 cells and HCV+ HepG2 cells were cocultured for 20 hr in microwells with the HCV TCR 1073 T cells. The production of IFN-γ and IL-2 were measured by ELISA. (0.06 MB TIF) [file ppat.1001018.s003.tif]

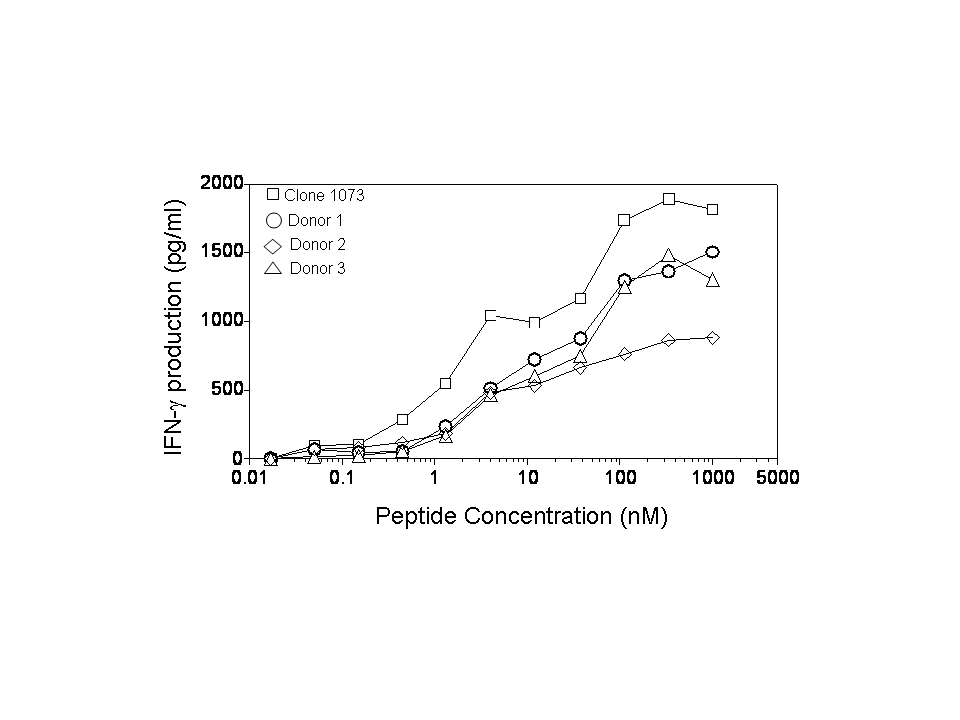

Supplement: Figure S4 — Relative avidity of HCV TCR 1073-transduced T cells. The functional avidity of HCV TCR transduced PBL-derived normal T cells was compared to the parent HCV NS3:1073:1081 T cell clone using interferon-γ release assays. T2 cells were loaded for 2 hr with varied concentrations of HCV NS3 1073–1081 peptide (0.01–1000 nM). These peptide-loaded T2 cells were cocultured for 20 hr in microwells with three different HCV TCR transduced T cell cultures or the parent HCV 1073-reactive T cell clone. The amount of interferon-γ produced was measured by ELISA. (0.06 MB TIF) [file ppat.1001018.s004.tif]
